# Supplementary material for: Implementation of multiparticle quantum speed limits on observables
Source: Sci Adv. 2025 Oct 31;11(44):eady0497. doi: 10.1126/sciadv.ady0497 (PMC13142044; doi:10.1126/sciadv.ady0497)
Supplement: Supplementary file 1 — Sections S1 to S7 Figs. S1 to S11 [file sciadv.ady0497_sm.pdf]

Science Advances
AAAS

Supplementary Materials for

Implementation of multiparticle quantum speed limits on observables

Rui-Heng Miao *et al.*

Corresponding author: Zhao-Di Liu, zdliu@ustc.edu.cn; Chuan-Feng Li, cfli@ustc.edu.cn

Sci. Adv. **11**, eady0497 (2025)
DOI: 10.1126/sciadv.ady0497

This PDF file includes:

Sections S1 to S7
Figs. S1 to S11

S1 The tighter quantum speed limits

Here, we introduce a more refined approach to achieving both a tighter upper bound and a new lower bound that involves splitting each operator and physical quantity into coherent and incoherent components (70). The density matrix ρ is decomposed into its eigenvalues and eigenstates as:

$$\rho = \sum_{j}^{2^N} p_j \left|j\right\rangle \left\langle j\right| . \tag{S1}$$

The observable A can be expressed in terms of the eigenstates $|j\rangle$, where the non-diagonal terms of the matrix represent the coherent component and the diagonal terms represent the incoherent component of observable A.

$$A_C = \sum_{j\neq k}^{2^N} \left|j\right\rangle \left\langle j\right| A \left|k\right\rangle \left\langle k\right| , \tag{S2}$$

$$A_I = \sum_{j}^{2^N} \left|j\right\rangle \left\langle j\right| A \left|j\right\rangle \left\langle j\right| . \tag{S3}$$

The standard deviations of the observable A are:

$$\Delta A = \sqrt{\langle A^2\rangle - \langle A\rangle^2}, \tag{S4}$$

$$\Delta A_C = \sqrt{\langle A_C^2\rangle - \langle A_C\rangle^2}, \tag{S5}$$

$$\Delta A_I = \sqrt{\langle A_I^2\rangle - \langle A_I\rangle^2}. \tag{S6}$$

Then, the coherent quantum speed is given by A_C, and the incoherent quantum speed is given by A_I, namely:

$$|\dot{a}_C| = \left|\mathrm{Tr}\left[\frac{\mathrm{d}\rho}{\mathrm{d}(l/\lambda)} A_C\right]\right|, \tag{S7}$$

$$|\dot{a}_I| = \left|\mathrm{Tr}\left[\frac{\mathrm{d}\rho}{\mathrm{d}(l/\lambda)} A_I\right]\right|. \tag{S8}$$

Similarly, coherent quantum Fisher information and incoherent quantum Fisher information are given by (64):

$$\mathbb{I}^F = 2 \sum_{jk:p_j+p_k>0}^{2^N} \frac{\left|\left\langle j\left|\frac{\mathrm{d}\rho(l)}{\mathrm{d}(l/\lambda)}\right|k\right\rangle\right|^2}{p_j+p_k}, \tag{S9}$$

$$\mathbb{I}_C^F = 2 \sum_{j\neq k:p_j+p_k>0}^{2^N} \frac{\left|\left\langle j\left|\frac{\mathrm{d}\rho(l)}{\mathrm{d}(l/\lambda)}\right|k\right\rangle\right|^2}{p_j+p_k}, \tag{S10}$$

$$\mathbb{I}_I^F = \sum_{j:p_j>0}^{2^N} \frac{\left|\left\langle j\left|\frac{\mathrm{d}\rho(l)}{\mathrm{d}(l/\lambda)}\right|j\right\rangle\right|^2}{p_j} = \sum_{j:p_j>0}^{2^N} p_j \left[\frac{\mathrm{d}}{\mathrm{d}(l/\lambda)} \ln p_j\right]^2. \tag{S11}$$

The quantum speed can also be separated into the coherent component $|\dot{a}_C|$ and the incoherent component $|\dot{a}_I|$, satisfying the relations $|\dot{a}_C| \leq \Delta A_C\sqrt{\mathbb{I}_C^F}$, $|\dot{a}_I| \leq \Delta A_I\sqrt{\mathbb{I}_I^F}$. As the total quantum speed is given by $|\dot{a}| = |\dot{a}_C + \dot{a}_I|$, thus, the quantum speed satisfies the following bounds:

$$|\dot{a}| \leq \min\left\{|\dot{a}_C| + \Delta A_I\sqrt{\mathbb{I}_I^F},\ |\dot{a}_I| + \Delta A_C\sqrt{\mathbb{I}_C^F}\right\}, \tag{S12}$$

$$|\dot{a}| \geq \max\left\{|\dot{a}_C| - \Delta A_I\sqrt{\mathbb{I}_I^F},\ |\dot{a}_I| - \Delta A_C\sqrt{\mathbb{I}_C^F}\right\}. \tag{S13}$$

Or, the quantum speed limits can be expressed as:

$$\max(b_{CI}^-, b_{IC}^-) \leq |\dot{a}| \leq \min(b_{CI}^+, b_{IC}^+). \tag{S14}$$

Here, $b_{mn}^{(\pm)} = |\dot{a}_m| \pm \Delta A_n\sqrt{\mathbb{I}_n^F}$. These are the tighter upper bound and new lower bound of quantum speed on the observable A. The only condition is that ρ is a density matrix which conforms to the quantum physics and A is a fixed observable, these bounds are independent of a specific evolution form (such as Schrödinger equation, Lindblad master equation).

S2 Detail evolution in our system

The initial total state of the N-particle open sysytem as:

$$|\psi_{SE}(0)\rangle = \sum_{i_1,i_2,\cdots,i_N}^{\{H,V\}^N} \iint\cdots\int \mathrm{d}\omega_1\mathrm{d}\omega_2\cdots\mathrm{d}\omega_N \Xi(i_1,\omega_1,i_2,\omega_2,\cdots,i_N,\omega_N,0)\,|i_1,\omega_1\rangle\otimes|i_2,\omega_2\rangle\otimes\cdots\otimes|i_N,\omega_N\rangle\,. \tag{S15}$$

Here $\Xi(i_1,\omega_1,i_2,\omega_2,\cdots,i_N,\omega_N,0)$ are the coefficients.

The initial density matrix of the polarization system is as follows:

$$\rho(0) = \mathrm{Tr}_E\left[|\psi_{SE}(0)\rangle\langle\psi_{SE}(0)|\right] = \iint\cdots\int \mathrm{d}\omega_1\mathrm{d}\omega_2\cdots\mathrm{d}\omega_N\ \langle\omega_1,\omega_2,\cdots,\omega_N|\psi_{SE}(0)\rangle\langle\psi_{SE}(0)|\omega_1,\omega_2,\cdots,\omega_N\rangle\,. \tag{S16}$$

After passing through a birefringent quartz crystal with an optic axis aligned in the $|H\rangle$ direction with a length L, the final total state of the open system is as follows:

$$\begin{aligned}|\psi_{SE}(L)\rangle =& \sum_{i_1,i_2,\cdots,i_N}^{\{H,V\}^N} \iint\cdots\int \mathrm{d}\omega_1\mathrm{d}\omega_2\cdots\mathrm{d}\omega_N \exp\left[\mathrm{i}\frac{n_{i_1}(\omega_1)\omega_1+n_{i_2}(\omega_2)\omega_2+\cdots+n_{i_N}(\omega_N)\omega_N}{c}L\right] \\ &\cdot\Xi(i_1,\omega_1,i_2,\omega_2,\cdots,i_N,\omega_N,0)\,|i_1,\omega_1\rangle\otimes|i_2,\omega_2\rangle\otimes\cdots\otimes|i_N,\omega_N\rangle \\ =& \sum_{i_1,i_2,\cdots,i_N}^{\{H,V\}^N} \iint\cdots\int \mathrm{d}\omega_1\mathrm{d}\omega_2\cdots\mathrm{d}\omega_N \kappa(i_1,\omega_1,i_2,\omega_2,\cdots,i_N,\omega_N,L)\,|i_1,\omega_1\rangle\otimes|i_2,\omega_2\rangle\otimes\cdots\otimes|i_N,\omega_N\rangle\,.\end{aligned} \tag{S17}$$

Here $\kappa(i_1,\omega_1,i_2,\omega_2,\cdots,i_N,\omega_N,L) = \exp\left[\mathrm{i}\frac{n_{i_1}(\omega_1)\omega_1+n_{i_2}(\omega_2)\omega_2+\cdots+n_{i_N}(\omega_N)\omega_N}{c}L\right]\Xi(i_1,\omega_1,i_2,\omega_2,\cdots,i_N,\omega_N,0)$. For polarization, $|H\rangle$ and $|V\rangle$ correspond to the extraordinary refractive index n_e and the ordinary refractive index n_o, respectively. To simplify the evolution equation, we define the optical path difference between H polarization and V polarization as $l = (\bar{n}_H-\bar{n}_V)L = (\bar{n}_e-\bar{n}_o)L$; this is used in our experiments to express the crystal length instead of L. Here, $\bar{n}_H=\bar{n}_e$ and $\bar{n}_V=\bar{n}_o$ are the average refractive indices of the extraordinary and ordinary rays, respectively. Throughout this paper, we express l in units of λ. Here, λ is the center wavelength. Therefore, the final total state of the open system is as follows:

$$|\psi_{SE}(l)\rangle = \sum_{i_1,i_2,\cdots,i_N}^{\{H,V\}^N} \iint\cdots\int \mathrm{d}\omega_1\mathrm{d}\omega_2\cdots\mathrm{d}\omega_N \Xi(i_1,\omega_1,i_2,\omega_2,\cdots,i_N,\omega_N,l)\,|i_1,\omega_1\rangle\otimes|i_2,\omega_2\rangle\otimes\cdots\otimes|i_N,\omega_N\rangle\,. \tag{S18}$$

Here $\Xi(i_1,\omega_1,i_2,\omega_2,\cdots,i_N,\omega_N,l) = \exp\left[\mathrm{i}\frac{n_{i_1}(\omega_1)\omega_1+n_{i_2}(\omega_2)\omega_2+\cdots+n_{i_N}(\omega_N)\omega_N}{(\bar{n}_H-\bar{n}_V)c}l\right]\Xi(i_1,\omega_1,i_2,\omega_2,\cdots,i_N,\omega_N,0)$.

If we regard the optical path difference between H polarization and V polarization l as the evolution time, the total Hamiltonian is as follows:

$$H_{\mathrm{SE}} = H_0\otimes I_{SE}^{\otimes(N-1)} + I_{SE}\otimes H_0\otimes I_{SE}^{\otimes(N-2)} + \cdots + I_{SE}^{\otimes(N-1)}\otimes H_0. \tag{S19}$$

Here $H_0 = \int\mathrm{d}\omega\left[\frac{n_H(\omega)\omega}{(\bar{n}_H-\bar{n}_V)c}|H,\omega\rangle\langle H,\omega| + \frac{n_V(\omega)\omega}{(\bar{n}_H-\bar{n}_V)c}|V,\omega\rangle\langle V,\omega|\right]$ is the Hamiltonian on single-particle open system. $\bar{n}_H$ and $\bar{n}_V$ are the average refractive indices of the H polarization (extraordinary rays) and V polarization (ordinary rays), respectively. $I_{SE}^{\otimes N}$ is the direct product of N identity matrices, for example, $I_{SE}^{\otimes 2} = I_{SE}\otimes I_{SE}$. I_{SE} is the identity matrix of single-particle open systems.

For the center frequency $\bar{\omega}$, $\frac{n_H(\omega)-\bar{n}}{\bar{n}_H-\bar{n}_V} = -\frac{n_V(\omega)-\bar{n}}{\bar{n}_H-\bar{n}_V} = \frac{1}{2}$. Here, $\bar{n} = (\bar{n}_H+\bar{n}_V)/2$. So the Hamiltonian of the single-particle open system with single frequency environment $\iint\cdots\int \mathrm{d}\omega_1\mathrm{d}\omega_2\cdots\mathrm{d}\omega_N\delta(\omega_1-\bar{\omega})\delta(\omega_2-\bar{\omega})\cdots\delta(\omega_N-\bar{\omega})\,|\omega_1,\omega_2,\cdots,\omega_N\rangle$ is:

$$\begin{aligned}H_0 =& \int\mathrm{d}\omega\left\{\frac{[n_H(\omega)-\bar{n}]\,\bar{\omega}}{(\bar{n}_H-\bar{n}_V)c}|H,\omega\rangle\langle H,\omega| + \frac{[n_V(\omega)-\bar{n}]\,\bar{\omega}}{(\bar{n}_H-\bar{n}_V)c}|V,\omega\rangle\langle V,\omega|\right\} \\ =& \frac{\omega}{2c}\left(|H\rangle\langle H| + |V\rangle\langle V|\right)\otimes\int\mathrm{d}\omega\,|\omega\rangle\langle\omega|\,. \\ =& \frac{\pi}{\lambda}\sigma_z\otimes I_E.\end{aligned} \tag{S20}$$

Here, δ is the Dirac delta function. I_E is the identity matrix of the single-particle frequency environment. σ_z is a Pauli

matrix, namely:

$$\sigma_x = \begin{pmatrix} 0 & 1 \\ 1 & 0 \end{pmatrix}, \tag{S21}$$

$$\sigma_y = \begin{pmatrix} 0 & -\mathrm{i} \\ \mathrm{i} & 0 \end{pmatrix}, \tag{S22}$$

$$\sigma_z = \begin{pmatrix} 1 & 0 \\ 0 & -1 \end{pmatrix}. \tag{S23}$$

The Hamiltonian of the single-particle polarization system with the single frequency environment is σ_z, so the Hamiltonian of the N-particle polarization system with the single frequency environment is:

$$H = \frac{\pi}{\lambda}\left[\sigma_z \otimes I^{\otimes(N-1)} + I \otimes \sigma_z \otimes I^{\otimes(N-2)} + \cdots + I^{\otimes(N-1)} \otimes \sigma_z\right]. \tag{S24}$$

Here, I is the identity matrix in single-particle polarization systems. As we can see, the evolution of the photon pair is the phase delay evolution in the σ_z direction.

The density matrix of the polarization system is as follows:

$$\rho(l) = \mathrm{Tr}_E\left[|\psi_{SE}(l)\rangle\langle\psi_{SE}(l)|\right]. \tag{S25}$$

S3 The speedup ratio among single-particle, product N-particle, and entangled N-particle qubits system

S3.1 Excluding mixed states

Assuming the initial density matrix is $\rho(0) = \sum_i^{2^N} p_i |\psi_i(0)\rangle\langle\psi_i(0)|$, which is a mixed density matrix. Here, $p_i \geq 0$, $\sum_i^{2^N} p_i = 1$ and $\langle\psi_i(0)|\psi_j(0)\rangle = \delta_{ij}$. Here $\delta_{ij} = 1$ if $i = j$ and $\delta_{ij} = 0$ if $i \neq j$. After evolution time l, the density matrix is $\rho(l) = \sum_i^{2^N} p_i |\psi_i(l)\rangle\langle\psi_i(l)|$ and $\langle\psi_i(l)|\psi_j(l)\rangle = \delta_{ij}$. The quantum speed is as follows:

$$|\dot{a}| = \left|\mathrm{Tr}\left[\frac{\mathrm{d}\rho}{\mathrm{d}(l/\lambda)}A\right]\right| = \left|\sum_i^{2^N} p_i \mathrm{Tr}\left[\frac{\mathrm{d}|\psi_i(l)\rangle\langle\psi_i(l)|}{\mathrm{d}(l/\lambda)}A\right]\right| \leq \max_i \left|\mathrm{Tr}\left[\frac{\mathrm{d}|\psi_i(l)\rangle\langle\psi_i(l)|}{\mathrm{d}(l/\lambda)}A\right]\right|. \tag{S26}$$

This quantum speed must be smaller than or equal to the quantum speed with a pure initial state. So it's unnecessary to consider the mixed states and complex environment during the complete evolution process if we want to reach the maximum quantum speed.

S3.2 The Hamiltonian and evolution

From Eq. (S24), we obtain the Hamiltonian for the N-qubit polarization system H. For the pure initial state $\rho(0) = |\psi(0)\rangle\langle\psi(0)|$, $U = \mathrm{e}^{\mathrm{i}Hl}$, $\rho(l) = U\rho(0)U^\dagger = |\psi(l)\rangle\langle\psi(l)|$ which is also pure state. The eigenstates of $\rho(l)$ are denoted as $\{|j\rangle\} = \{|\psi(l)\rangle, |\perp_2\rangle, |\perp_3\rangle, \cdots, |\perp_{2^N}\rangle\}$, the last $2^N - 1$ states are temporary symbols which won't appear in the final result. Eigenvalues are $p_1 = 1$, $p_2 = p_3 = \cdots = p_{2^N} = 0$.

For the derivatives of U, H is a diagonal matrix, fortunately, so we can simplify it to:

$$\begin{aligned}\frac{\mathrm{d}U}{\mathrm{d}(l/\lambda)} =& \frac{\mathrm{d}\mathrm{e}^{\mathrm{i}\pi\frac{l}{\lambda}\left[\sigma_z\otimes I^{\otimes(N-1)} + I\otimes\sigma_z\otimes I^{\otimes(N-2)} + I^{\otimes 2}\otimes\sigma_z\otimes I^{\otimes(N-3)} + \cdots + I^{\otimes(N-1)}\otimes\sigma_z\right]}}{\mathrm{d}(l/\lambda)} \\ =& \mathrm{i}\pi\left[\sigma_z \otimes I^{\otimes(N-1)} + I \otimes \sigma_z \otimes I^{\otimes(N-2)} + I^{\otimes 2} \otimes \sigma_z \otimes I^{\otimes(N-3)} + \cdots + I^{\otimes(N-1)} \otimes \sigma_z\right] U \\ =& WU.\end{aligned} \tag{S27}$$

Here, $W = \mathrm{i}\pi\left[\sigma_z \otimes I^{\otimes(N-1)} + I \otimes \sigma_z \otimes I^{\otimes(N-2)} + I^{\otimes 2} \otimes \sigma_z \otimes I^{\otimes(N-3)} + \cdots + I^{\otimes(N-1)} \otimes \sigma_z\right] = \mathrm{i}\lambda H$ is a diagonal matrix and $W^\dagger = -W$, we can also derivation that $\frac{\mathrm{d}U^\dagger}{\mathrm{d}(l/\lambda)} = U^\dagger W^\dagger = -U^\dagger W$.

We are ready to calculate the rate of change of the density matrix, namely:

$$\frac{\mathrm{d}\rho(l)}{\mathrm{d}(l/\lambda)} = W\rho(l) - \rho(l)W. \tag{S28}$$

The quantum Fisher information is based on the rate of change of the density matrix; the incoherent quantum Fisher information is (70):

$$\mathbb{I}_I^F=\sum_{j:p_j>0}^{2^N}\frac{\left|\left\langle j\left|\frac{\mathrm{d}\rho(l)}{\mathrm{d}(l/\lambda)}\right|j\right\rangle\right|^2}{p_j}=\frac{\left|\left\langle\psi(l)\left|\frac{\mathrm{d}\rho(l)}{\mathrm{d}(l/\lambda)}\right|\psi(l)\right\rangle\right|^2}{p_1}=\frac{\left|\langle\psi(l)|W|\psi(l)\rangle-\langle\psi(l)|W|\psi(l)\rangle\right|^2}{p_1}=0. \tag{S29}$$

The coherent quantum Fisher information is (70):

$$\begin{aligned}\mathbb{I}_C^F=&2\sum_{j\neq k:p_j+p_k>0}^{2^N}\frac{\left|\left\langle j\left|\frac{\mathrm{d}\rho(l)}{\mathrm{d}(l/\lambda)}\right|k\right\rangle\right|^2}{p_j+p_k}\\ =&\frac{4}{p_1}\langle\psi(l)|\frac{\mathrm{d}\rho(l)}{\mathrm{d}(l/\lambda)}\left(\sum_{j=2}^{2^N}|\perp_j\rangle\langle\perp_j|\right)\frac{\mathrm{d}\rho^\dagger(l)}{\mathrm{d}(l/\lambda)}|\psi(l)\rangle\\ =&4\langle\psi(l)|\frac{\mathrm{d}\rho(l)}{\mathrm{d}(l/\lambda)}\left(I-|\psi(l)\rangle\langle\psi(l)|\right)\frac{\mathrm{d}\rho(l)}{\mathrm{d}(l/\lambda)}|\psi(l)\rangle\\ =&4\langle\psi(l)|\frac{\mathrm{d}\rho(l)}{\mathrm{d}(l/\lambda)}\frac{\mathrm{d}\rho(l)}{\mathrm{d}(l/\lambda)}|\psi(l)\rangle-4\langle\psi(l)|\frac{\mathrm{d}\rho(l)}{\mathrm{d}(l/\lambda)}|\psi(l)\rangle\langle\psi(l)|\frac{\mathrm{d}\rho(l)}{\mathrm{d}(l/\lambda)}|\psi(l)\rangle\\ =&4\langle\psi(l)|\frac{\mathrm{d}\rho(l)}{\mathrm{d}(l/\lambda)}\frac{\mathrm{d}\rho(l)}{\mathrm{d}(l/\lambda)}|\psi(l)\rangle\\ =&4\langle\psi(l)|[W\rho(l)-\rho(l)W][W\rho(l)-\rho(l)W]|\psi(l)\rangle\\ =&4\langle\psi(l)|[W\rho(l)W-WW]|\psi(l)\rangle.\end{aligned} \tag{S30}$$

As H, W, and U have the same eigenstates, they can exchange product orders. The above formula can be simplified. $\mathbb{I}_C^F$ is constant as the evolution time l changes, only related to the initial state, namely:

$$\begin{aligned}\mathbb{I}_C^F=&4\langle\psi(l)|[W\rho(l)W-WW]|\psi(l)\rangle\\ =&4\langle\psi(0)|U^\dagger[WU\rho(0)U^\dagger W-WW]U|\psi(0)\rangle\\ =&4\langle\psi(0)|[W\rho(0)W-WW]|\psi(0)\rangle\\ =&4\lambda^2\langle\psi(0)|HH|\psi(0)\rangle-4\lambda^2\langle\psi(0)|H|\psi(0)\rangle\langle\psi(0)|H|\psi(0)\rangle\\ =&4\lambda^2\Delta H^2.\end{aligned} \tag{S31}$$

$$\sqrt{\mathbb{I}_C^F}=2\lambda\Delta H. \tag{S32}$$

The eigenstates of H are denoted as $\{|e_1\rangle,\ |e_2\rangle,\cdots,\ |e_{2^N}\rangle\}$, with eigenvalues $E_1,E_2,\cdots E_{2^N}$. Initial state can be decompose to $|\psi(0)\rangle=\sum_i^{2^N}\alpha_i|e_i\rangle$, then:

$$\sqrt{\mathbb{I}_C^F}=2\lambda\sqrt{\sum_i^{2^N}|\alpha_i|^2E_i^2-\left(\sum_i^{2^N}|\alpha_i|^2E_i\right)^2}. \tag{S33}$$

Note that $\mathbb{I}_C^F$ is only related to the energy distribution of the initial state; phase contributes nothing to the quantum Fisher information, so modulating the phase of each result in the following paper is ok, we only show the simplest results.

In single-particle qubit systems, the eigenvalues of H is $+\frac{\pi}{\lambda},-\frac{\pi}{\lambda}$. In the Bernoulli distribution (0-1 distribution), to achieve maximum variance, we must evenly distribute the initial state to both energy levels, so the best initial state is:

$$|\psi(0)\rangle=(|H\rangle+|V\rangle)/\sqrt{2}=|+\rangle. \tag{S34}$$

$$\sqrt{\mathbb{I}_C^F}=2\pi. \tag{S35}$$

In product N-particle qubits systems, ΔH^2 of the N-particle system is a sum of the variance of the Hamiltonian of each single-particle system, so we should evenly distribute the initial state to both energy levels of every particle, namely:

$$|\psi(0)\rangle=|+\rangle\otimes|+\rangle\otimes\cdots\otimes|+\rangle=|+\rangle^{\otimes N}. \tag{S36}$$

$$\sqrt{\mathbb{I}_C^F}=2\sqrt{N}\pi. \tag{S37}$$

In entangled N-particle qubits systems, the maximum and minimum eigenvalues of H are $+\frac{N\pi}{\lambda}$ and $-\frac{N\pi}{\lambda}$. We also evenly distributed the initial state to the highest and lowest levels, namely:

$$|\psi(0)\rangle = \left(|H\rangle^{\otimes N} + |V\rangle^{\otimes N}\right)/\sqrt{2}. \tag{S38}$$

$$\sqrt{\mathbb{I}_C^F} = 2N\pi. \tag{S39}$$

The ratio of $\sqrt{\mathbb{I}_C^F}$ among single-particle qubit system, product N-particle qubits system, and entangled N-particle qubits system is $1 : \sqrt{N} : N$.

Only in entangled N-particle qubits systems, we can distribute the initial state to different levels with the same energy, which will lead to zero coherent quantum Fisher information, for example:

$$|\psi(0)\rangle = (|HV\rangle + |VH\rangle)/\sqrt{2} = \left|\Psi^+\right\rangle \quad \text{or such as} \quad (|HHV\rangle + |HVH\rangle + |VHH\rangle)/\sqrt{3}. \tag{S40}$$

$$\sqrt{\mathbb{I}_C^F} = 0. \tag{S41}$$

S3.3 The quantum speed

For the upper bound in our paper, we should split the arbitrary observable A into coherent and incoherent parts. The coherent part is as follows (70):

$$A_C = \sum_{j\neq k}^{2^N} |j\rangle\langle j| A |k\rangle\langle k|. \tag{S42}$$

$$\mathrm{Tr}\left[\rho(l)A_C\right] = \langle\psi(l)| A_C |\psi(l)\rangle = 0. \tag{S43}$$

$$\begin{aligned}\mathrm{Tr}\left[\rho(l)A_C^2\right] &= \langle\psi(l)| A_C^2 |\psi(l)\rangle \\ &= \langle\psi(l)| A \left(\sum_{j=2}^{2^N} |\perp_j\rangle\langle\perp_j|\right) A |\psi(l)\rangle \\ &= \langle\psi(l)| A \left(I - |\psi(l)\rangle\langle\psi(l)|\right) A |\psi(l)\rangle \\ &= \langle\psi(l)| A |\psi(l)\rangle - \langle\psi(l)| A |\psi(l)\rangle\langle\psi(l)| A |\psi(l)\rangle \\ &= a - a^2.\end{aligned} \tag{S44}$$

$$\Delta A_C = \sqrt{\mathrm{Tr}\left[\rho(l)A_C^2\right] - \left(\mathrm{Tr}\left[\rho(l)A_C\right]\right)^2} = \sqrt{a}\sqrt{1-a}. \tag{S45}$$

ΔA_C will reach the maximum value $\Delta A_C = 1/2$ when $a = 1/2$. The expectation values a of all initial states among Eq. (S34), Eq. (S36) and Eq. (S38) move continuously from 1 to 0 as the evolution time l changes if we set $A = \rho(0)$, so all of them can find a appropriate l as $a = 1/2$. At the same time, the incoherent part is as follows (70):

$$A_I = \sum_{j}^{2^N} |j\rangle\langle j| A |j\rangle\langle j| = |\psi(l)\rangle\langle\psi(l)| A |\psi(l)\rangle\langle\psi(l)| + \sum_{j=2}^{2^N} |\perp_j\rangle\langle\perp_j| A |\perp_j\rangle\langle\perp_j|. \tag{S46}$$

$$\mathrm{Tr}\left[\rho(l)A_I\right] = \langle\psi(l)| A_I |\psi(l)\rangle = \langle\psi(l)| A |\psi(l)\rangle = a. \tag{S47}$$

$$\mathrm{Tr}\left[\rho(l)A_I^2\right] = \langle\psi(l)| A_I^2 |\psi(l)\rangle = \langle\psi(l)| A |\psi(l)\rangle\langle\psi(l)| A |\psi(l)\rangle = a^2. \tag{S48}$$

$$\Delta A_I = \sqrt{\mathrm{Tr}\left[\rho(l)A_I^2\right] - \left(\mathrm{Tr}\left[\rho(l)A_I\right]\right)^2} = 0. \tag{S49}$$

Then the incoherent quantum speed is:

$$\dot{a}_I = \mathrm{Tr}\left[\frac{\mathrm{d}\rho(l)}{\mathrm{d}(l/\lambda)}A_I\right] = \mathrm{Tr}\left[A_I W\rho(l) - \rho(l)WA_I\right] = \langle\psi(l)|A|\psi(l)\rangle\langle\psi(l)|W|\psi(l)\rangle - \langle\psi(l)|W|\psi(l)\rangle\langle\psi(l)|A|\psi(l)\rangle = 0. \tag{S50}$$

Then the coherent quantum speed is:

$$\dot{a}_C = \dot{a} - \dot{a}_I = \mathrm{Tr}\left[AW\rho(l) - \rho(l)WA\right] = \langle\psi(l)|\psi(0)\rangle\langle\psi(0)|W|\psi(l)\rangle + \langle\psi(l)\left|W^\dagger\right|\psi(0)\rangle\langle\psi(0)|\psi(l)\rangle. \tag{S51}$$

Comparing with the quantum Fisher information, we can derive that:

$$|\dot{a}_C| = \left|\mathrm{Tr}\left[\frac{\mathrm{d}\rho(l)}{\mathrm{d}(l/\lambda)}A\right]\right| = \lambda\left|\mathrm{Tr}\left[\frac{\mathrm{d}\rho(l)}{\mathrm{d}(l)}A\right]\right| = \lambda\left|\mathrm{Tr}\left\{\left[H\rho(l) - \rho(l)H\right]A\right\}\right| \leq 2\lambda\Delta A\Delta H = \Delta A_C\sqrt{\mathbb{I}_C^F}. \tag{S52}$$

S3.4 The upper bound of the quantum speed

To sum up, the upper bound $|\dot{a}| \leq \min(b^+_{CI}, b^+_{IC})$ (70) can be simplified when the frequency distribution is narrow and the initial state is pure as:

$$|\dot{a}| \leq \sqrt{a}\sqrt{1-a}\sqrt{\mathbb{I}^F_C}. \tag{S53}$$

In this formula, $\sqrt{\mathbb{I}^F_C}$ decides the initial state, a decides the appropriate evolution time l.

In single-particle qubit systems, the best initial state is $|\psi(0)\rangle = |+\rangle$ with the best evolution time $l = \frac{\lambda}{4} + k\frac{\lambda}{2}$, $k \in \mathbb{Z}$, the maximum upper bound around all initial states and all evolution time is π.

In product N-particle qubits systems, the best initial state is $|\psi(0)\rangle = |+\rangle^{\otimes N}$ with the best evolution time $l = \pm\lambda \arccos(2^{(N-1)/N} - 1)/(2\pi) + k\lambda$, $k \in \mathbb{Z}$, the maximum upper bound around all initial states and all evolution time is $\sqrt{N}\pi$.

In entangled N-particle qubits systems, the best initial state is $|\psi(0)\rangle = \left(|H\rangle^{\otimes N} + |V\rangle^{\otimes N}\right)/\sqrt{2}$ with the best evolution time $l = \left(\frac{\lambda}{4} + k\frac{\lambda}{2}\right)/N$, $k \in \mathbb{Z}$, the maximum upper bound around all initial states and all evolution time is $N\pi$.

We can also find zero-speed states. Excluding trivial solutions (for example, $|\psi(0)\rangle = |H\rangle$), we can find some initial states such as $|\psi(0)\rangle = |\Psi^+\rangle$ or $|\psi(0)\rangle = (|HHV\rangle + |HVH\rangle + |VHH\rangle)/\sqrt{3}$ only in entangled systems. The maximum upper bounds around these initial states are 0, no matter what evolution time is as $\sqrt{\mathbb{I}^F_C} = 0$. So the quantum speed can only be zero, regardless of the observable. For nonunitary multiparticle systems, the quantum speeds are not strictly equal to 0, but only close to 0 because of the interference from the frequency environments.

S3.5 The lower bound of the quantum speed

The lower bound $\max(b^-_{CI}, b^-_{IC}) \leq |\dot{a}|$ (70) can also be simplified as:

$$b^-_{CI} = |\dot{a}_C| - \Delta A_I\sqrt{\mathbb{I}^F_I} = |\dot{a}|, \tag{S54}$$

$$b^-_{IC} = |\dot{a}_I| - \Delta A_C\sqrt{\mathbb{I}^F_C} = -2\sqrt{a}\sqrt{1-a}\lambda\Delta H. \tag{S55}$$

Then, the lower bound is:

$$\max(b^-_{CI}, b^-_{IC}) = |\dot{a}|. \tag{S56}$$

We can conclude that, if we do not consider mixed states, nonunitary noises, and complex environments during the complete evolution process, the lower bound of quantum speed limits can be tight. As shown in Fig. S4 (e-h, m-n) and Fig. S5 (e-h, m-n), the lower bounds of quantum speed limits are almost equal to the quantum speeds. We must point out here that if the above conditions are not met, such as the addition of nonunitary noises, the lower bounds of the quantum speed will be separated from the quantum speeds, as shown in Fig. S4 (o-p) and Fig. S5 (o-p).

S4 Multi-photon sources

S4.1 Two-photon sources from nonlinear crystals

The initial state of the two-particle frequency environment is:

$$|\psi_E(0)\rangle = \iint \mathrm{d}\omega_1 \mathrm{d}\omega_2 g_2(\omega_1, \omega_2)\,|\omega_1, \omega_2\rangle\,. \tag{S57}$$

When a type-I β-BBO crystal is pumped by a continuous-wave laser with a frequency distribution $\int \mathrm{d}\omega_p g_p(\omega_p)\,|\omega_p\rangle_p$, whose center wavelength is 404nm, the output is a negatively correlated frequency-entangled two-particle state, as shown in Fig. S1 (a) and expressed as:

$$g_2(\omega_1, \omega_2) = -\frac{\mathrm{i}\omega_p^2\chi^{(2)}_{\mathrm{eff}}}{k_p c} L_B g_p(\omega_p) \mathrm{sinc}\left[(k_1 + k_2 - k_p)\frac{L_B}{2}\right] \exp\left[\mathrm{i}\,(k_1 + k_2 - k_p)\frac{L_B}{2}\right] F(\omega_1, \omega_2). \tag{S58}$$

Here, ω_1 and ω_2 are the frequencies of the two photons, and the relation $\bar{\omega}_p \approx \bar{\omega}_1 + \bar{\omega}_2$ follows from the conservation of energy. $\chi^{(2)}_{\mathrm{eff}}$ represents the second-order nonlinear optical susceptibility. The wave vector is given by $k = n\omega/c$, where n is the refractive index of β-BBO, which accounts for both polarization and dispersion. The parameter $L_B = 0.5$mm represents

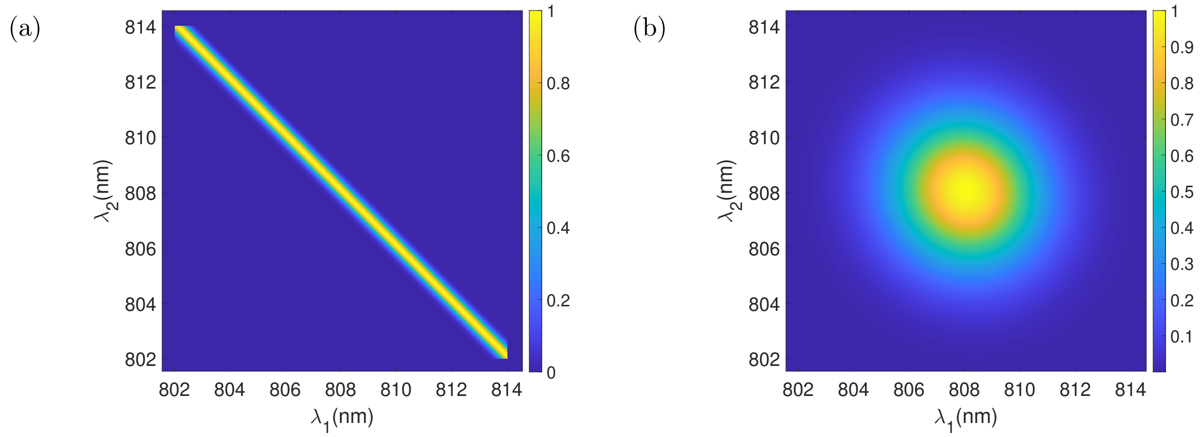

FIG. S1. **Frequency distribution of different two-photon sources. (a)** The amplitude of the initial state $|\psi_E(0)\rangle$ of the two-particle frequency correlated environment in our experiment. We use the corresponding wavelength λ instead of the frequency ω on the coordinate axes. It is a negatively correlated frequency-entangled state limited by 12 nm bandpass filters on both photons using a type-I β-BBO crystal pumped by a continuous-wave laser. **(b)** The amplitude of the initial state $|\psi_E(0)\rangle$ of the two-particle frequency correlated environment using a type-II β-BBO crystal pumped by a mode-locked femtosecond laser and 2nm Gaussian bandpass filters.

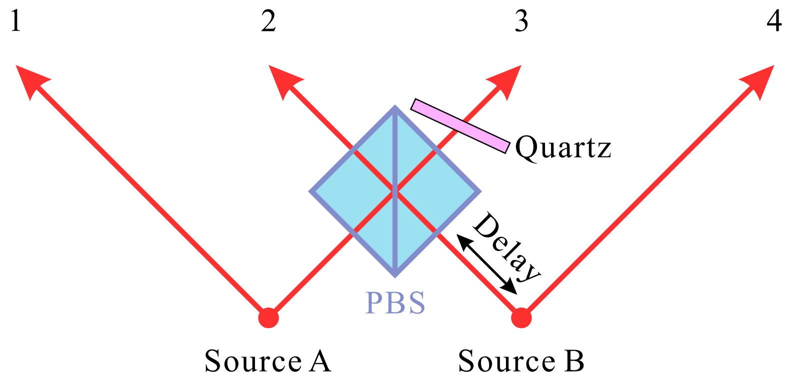

FIG. S2. **Diagrammatic sketch of experimental setup for frequency decorrelated four-photon source.** Each source generates one photon pair. A polarizing beam splitter (PBS) is used to interfere the second photon of source A and the first photon of source B. The delay is used to adjust the time overlap between two photon sources. The tilted quartz is used to adjust the time overlap between different polarizations.

the length of the β-BBO crystal. And the $F(\omega_1, \omega_2)$ represents 12 nm bandpass filters on both photons. In our experiment, we use this negatively correlated frequency-entangled source.

For frequency decorrelated two-particle sources, we can replace the 0.5mm type-I β-BBO crystal with a 0.2mm type-II β-BBO crystal, replace the continuous-wave laser with a mode-locked femtosecond laser, and replace the 12 nm bandpass filters with 2nm Gaussian bandpass filters. As shown in Fig. S1 (b), the frequency distribution is close to the direct product state on two photons.

The initial total state of the two-particle open system can be a product state between the polarization system and the frequency environment, namely:

$$\begin{aligned}|\psi_{SE}(0)\rangle &= |\psi(0)\rangle \otimes |\psi_E(0)\rangle \\ &= \sum_{i_1,i_2}^{\{H,V\}^2} \iint \mathrm{d}\omega_1 \mathrm{d}\omega_2 \xi_{i_1,i_2} g_2(\omega_1,\omega_2) |i_1,\omega_1\rangle \otimes |i_2,\omega_2\rangle \,. \end{aligned} \tag{S59}$$

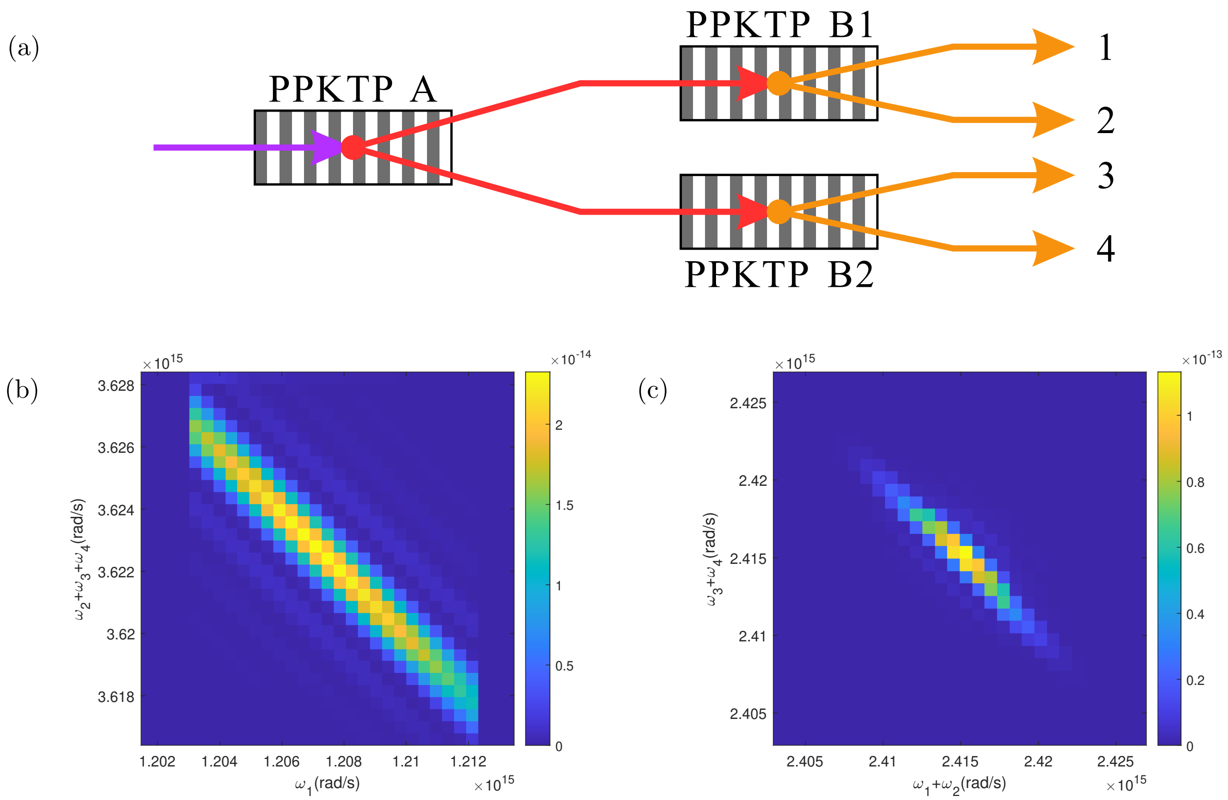

FIG. S3. **Frequency distribution of frequency correlated four-photon sources. (a)** Diagrammatic sketch of experimental setup for frequency correlated four-photon source. Each periodically poled potassium titanyl phosphate (PPKTP) generates one photon pair. PPKTP A is pumped by a 390nm mode-locked femtosecond laser, which will transfer the laser to 780nm photon pairs. Then each pair will be converted to four 1560nm photons by PPKTP B1 and B2. **(b)** Projection of four-particle frequency correlation on dimension ω_1 and dimension $\omega_2+\omega_3+\omega_4$. **(c)** Projection of four-particle frequency correlation on dimension $\omega_1+\omega_2$ and dimension $\omega_3+\omega_4$.

Here, the initial state of the two-particle polarization system is:

$$|\psi(0)\rangle = \xi_{HH}\,|HH\rangle + \xi_{HV}\,|HV\rangle + \xi_{VH}\,|VH\rangle + \xi_{VV}\,|VV\rangle\,. \tag{S60}$$

Here, ξ_{HH}, ξ_{HV}, ξ_{VH}, ξ_{VV} are the coefficients and $|\xi_{HH}|^2+|\xi_{HV}|^2+|\xi_{VH}|^2+|\xi_{VV}|^2=1$.

S4.2 Frequency decorrelated multi-photon sources

For four-particle sources, we can use another photon source to generate another photon pair, then use a polarizing beam splitter (PBS) to interfere the second photon of source A and the first photon of source B (74, 82, 83). As shown in Fig. S2, delay is used to adjust the time overlap between two photon sources; tilted quartz is used to adjust the time overlap between different polarizations. When we only collect four-photon coincidence signals, the initial total state is:

$$\begin{aligned}|\psi_{SE}(0)\rangle = \sum_{i_1,i_4}^{\{H,V\}^2}\iiiint \mathrm{d}\omega_1\mathrm{d}\omega_2\mathrm{d}\omega_3\mathrm{d}\omega_4\,&(\xi_{i_1,H}\,g_2(\omega_1,\omega_3)\xi_{H,i_4}\,g_2(\omega_2,\omega_4)\,|i_1,\omega_1\rangle\otimes|H,\omega_2\rangle\otimes|H,\omega_3\rangle\otimes|H,\omega_4\rangle\\ &-\xi_{i_1,V}\,g_2(\omega_1,\omega_2)\xi_{V,i_4}\,g_2(\omega_3,\omega_4)\,|i_1,\omega_1\rangle\otimes|V,\omega_2\rangle\otimes|V,\omega_3\rangle\otimes|i_4,\omega_4\rangle)\,.\end{aligned} \tag{S61}$$

We can see that, if we use frequency correlated two-photon sources, the polarization system and frequency environment would be entangled together, so we must use frequency decorrelated two-photon sources here as shown in Fig. S1 (b). When we consider the case of the product four-particle system, we set the quantum state of the polarization system for each photon pair to $|HH\rangle$, then the polarization state after PBS is $|HHHH\rangle$. And when we consider the case of the entangled four-particle system, we can achieve $|\Phi^+\rangle$ polarization state for each photon pair using two-layer β-BBO, then the polarization state after PBS is $(|HHHH\rangle - |VVVV\rangle)/\sqrt{2}$. We can use half-wave plates (HWPs) and quarter-wave plates (QWPs) to modify the polarization states to some specific quantum states such as $|++++\rangle$, $|PPPP\rangle$, $(|HHHH\rangle+|VVVV\rangle)/\sqrt{2}$, and $(|HVHV\rangle+|VHVH\rangle)/\sqrt{2}$.

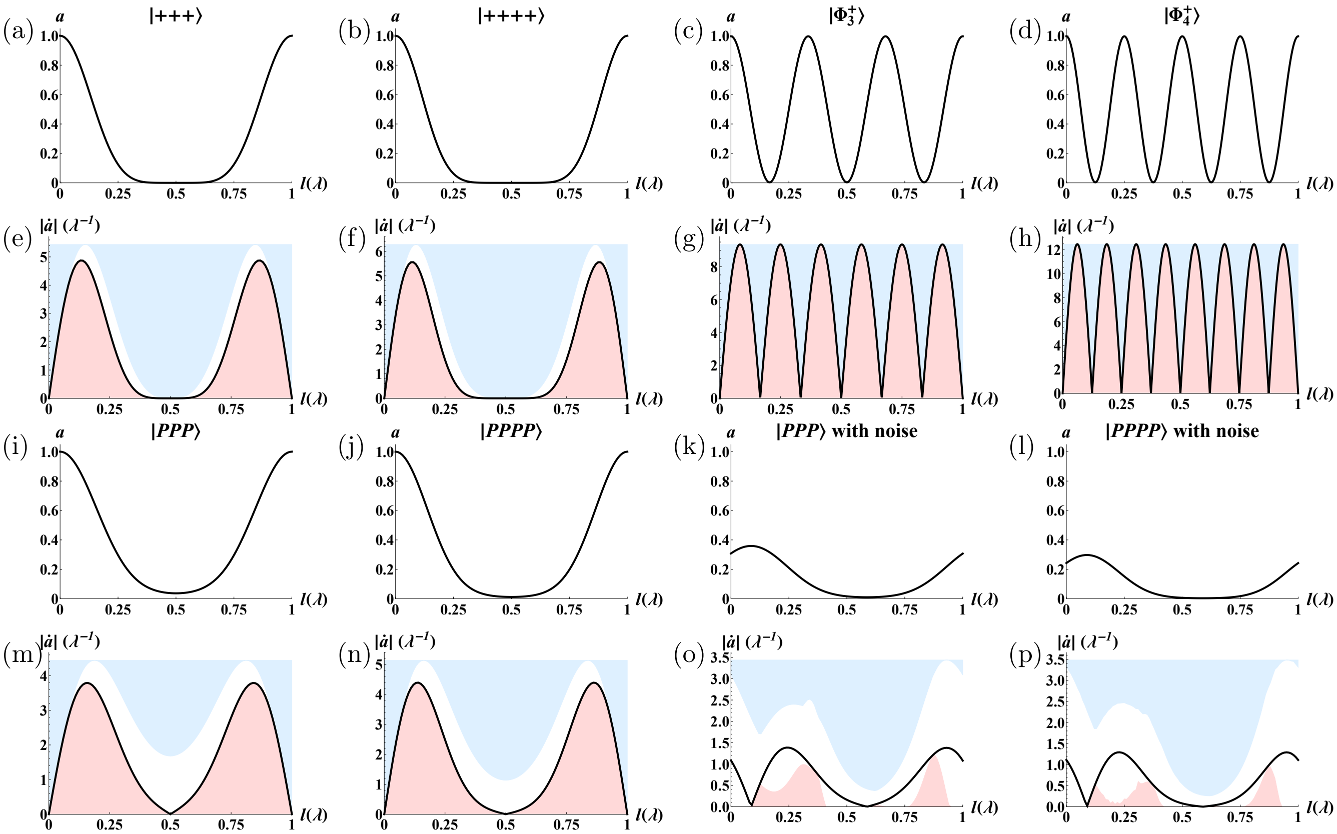

FIG. S4. **Quantum speed limits in three-particle and four-particle frequency decorrelated systems. (a-d, i-l)** The black line represents the expectation value a in theory. **(e-h, m-p)** represent quantum speeds $|\dot{a}|$ and speed limits on observables of the initial state. As the quantum speed $|\dot{a}| \geq 0$, subfigures are drawn from the y-axis 0. The black line represents the quantum speed $|\dot{a}|$ in theory. The blue and red areas indicate the forbidden areas beyond the upper bound and below the lower bound, respectively. Different column pairs represent different initial states, which are $|+++\rangle$, $|++++\rangle$, $\left|\Phi_3^+\right\rangle$, $\left|\Phi_4^+\right\rangle$, $|PPP\rangle$, $|PPPP\rangle$, $|PPP\rangle$, $|PPPP\rangle$, respectively. Here $\left|\Phi_3^+\right\rangle = (|HHH\rangle + |VVV\rangle)/\sqrt{2}$, $\left|\Phi_4^+\right\rangle = (|HHHH\rangle + |VVVV\rangle)/\sqrt{2}$. There are extra nonunitary noises after the evolution in (k, l, o, p).

By repeating the above operation, we can extend the number of particles to a larger even number N. For an odd number N-particle open system, we can just abandon the last photon from $N+1$-particle open system.

S4.3 Frequency correlated multi-photon sources

Since only photons of the same frequency can interfere on PBS, PBS multi-photon sources can only generate frequency decorrelated photons, which is not a good method for the research field of open systems. Here, we introduce another way to achieve multi-photon sources. Using the cascade of spontaneous parametric down-conversion crystals, we can generate a few photons whose frequency sum is almost constant, namely, frequency correlated multi-photon sources (86). Several three-photon experiments have been conducted using this method (84, 85, 87). Let us take Fig. S3 (a) as an example, periodically poled potassium titanyl phosphate (PPKTP) A will transfer a 390nm laser to 780nm photon pairs, then each photon pair will be converted to four 1560nm photons by PPKTP B1 and B2. The frequency distribution is:

$$
\begin{aligned}
g_4(\omega_1,\omega_2,\omega_3,\omega_4) = &-\frac{\mathrm{i}\omega_{pA}^2\chi_{\mathrm{eff},A}^{(2)}}{k_{pA}c}\frac{\mathrm{i}\omega_{pB1}^2\chi_{\mathrm{eff},B1}^{(2)}}{k_{pB1}c}\frac{\mathrm{i}\omega_{pB2}^2\chi_{\mathrm{eff},B2}^{(2)}}{k_{pB2}c}L_AL_{B1}L_{B2}g_{pA}(\omega_p)\mathrm{sinc}\left[(k_1+k_2-k_{pB1})\frac{L_{B1}}{2}\right]\cdot\\
&\mathrm{sinc}\left[(k_3+k_4-k_{pB2})\frac{L_{B2}}{2}\right]\mathrm{sinc}\left[(k_{pB1}+k_{pB2}-k_{pA})\frac{L_A}{2}\right]\exp\left[\mathrm{i}(k_1+k_2-k_{pB1})\frac{L_{B1}}{2}\right]\cdot\\
&\exp\left[\mathrm{i}(k_3+k_4-k_{pB2})\frac{L_{B2}}{2}\right]\exp\left[\mathrm{i}(k_{pB1}+k_{pB2}-k_{pA})\frac{L_A}{2}\right]F(\omega_1,\omega_2,\omega_3,\omega_4). \qquad \text{(S62)}
\end{aligned}
$$

Here, ω_1, ω_2, ω_3 and ω_4 are the frequencies of the four photons, ω_{pB1} is the frequency of the pump light of PPKTP B1, namely $\omega_{pB1} = \omega_1 + \omega_2$. Similarly, $\omega_{pB2} = \omega_3 + \omega_4$ and $\omega_{pA} = \omega_1 + \omega_2 + \omega_3 + \omega_4$. The relation $\bar{\omega}_{pA} \approx \bar{\omega}_1 + \bar{\omega}_2 + \bar{\omega}_3 + \bar{\omega}_4$

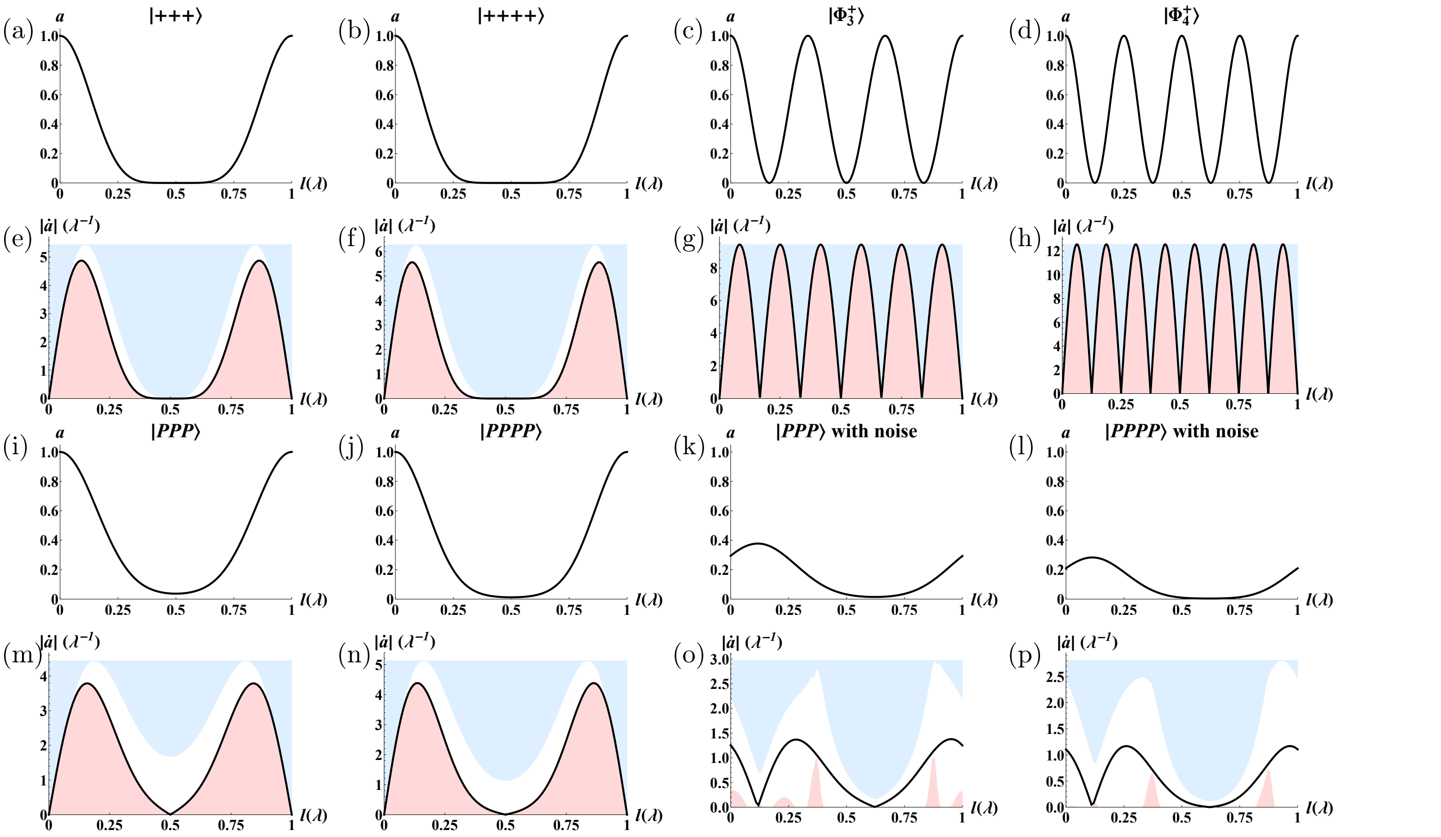

FIG. S5. **Quantum speed limits in three-particle and four-particle frequency correlated systems.** **(a-d, i-l)** The black line represents the expectation value a in theory. **(e-h, m-p)** represent quantum speeds $|\dot{a}|$ and speed limits on observables of the initial state. As the quantum speed $|\dot{a}| \geq 0$, subfigures are drawn from the y-axis 0. The black line represents the quantum speed $|\dot{a}|$ in theory. The blue and red areas indicate the forbidden areas beyond the upper bound and below the lower bound, respectively. Different column pairs represent different initial states, which are $|+++\rangle$, $|++++\rangle$, $|\Phi_3^+\rangle$, $|\Phi_4^+\rangle$, $|PPP\rangle$, $|PPPP\rangle$, $|PPP\rangle$, $|PPPP\rangle$, respectively. Here $|\Phi_3^+\rangle = (|HHH\rangle + |VVV\rangle)/\sqrt{2}$, $|\Phi_4^+\rangle = (|HHHH\rangle + |VVVV\rangle)/\sqrt{2}$. There are extra nonunitary noises after the evolution in (k, l, o, p).

follows from the conservation of energy. $\chi_{\text{eff}}^{(2)}$ represents the second-order nonlinear optical susceptibility. The wave vector is given by $k = n\omega/c$, where n is the refractive index of PPKTP, which accounts for both polarization and dispersion. The parameter $L_A = L_{B1} = L_{B2} = 0.2$mm represents the length of the PPKTP crystal. And the $F(\omega_1, \omega_2, \omega_3, \omega_4)$ represents 12 nm bandpass filters on four photons. As shown in Fig. S3 (b, c), we display two projections of the four-particle frequency correlation.

At this time, the total quantum state can be a product state between the polarization state and the frequency state. For the product polarization state, we can use type-0 PPKTP to generate state $|HH\rangle$ in each PPKTP, and the four-photon polarization state would be $|HHHH\rangle$. For the entangled polarization state, we can use type-0 two-layer PPKTP to generate state $|\Phi^+\rangle$ in each PPKTP, and the four-photon polarization state would be $(|HHHH\rangle + |VVVV\rangle)/\sqrt{2}$.

Note that this kind of frequency correlated four-photon sources can be extended to multi-photon sources (86), it is sufficient for discussing quantum speed limits.

S4.4 The quantum speed limits for three-particle and four-particle systems in theory

For frequency decorrelated multi-photon sources using PBS, we simulated the quantum speed limits in up to four-particle systems in theory. For systems with more particles, it would require a larger amount of memory to simulate the frequency distribution, which is difficult to achieve. The quantum speed limits in single-particle and two-particle systems have already been shown in Fig. 3 of the main letter with the experimental results. Here, as shown in Fig. S4, we display the quantum speed limits in three-particle and four-particle frequency decorrelated systems.

For frequency correlated multi-photon sources using cascade of spontaneous parametric down-conversion crystals, we can also simulate the quantum speed limits in up to four-particle systems in theory, as shown in Fig. S5.

Due to the short evolution time, in most cases, the deviations between quantum speed limits in frequency decorrelated

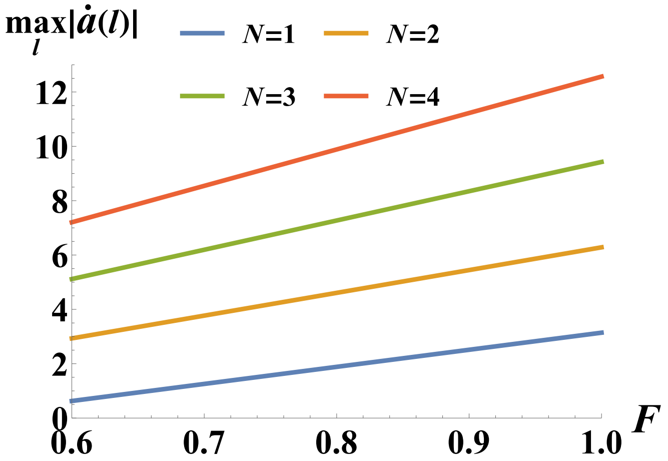

FIG. S6. **Fidelity F vs maximum quantum speed $\max_l |\dot{a}(l)|$ in multi-photon experiments.**

systems and frequency correlated systems are less than the calculation errors. However, the σ_x nonunitary noise can uncover the differences between these two kinds of frequency environments, as shown in Fig. S4 (o, p) and Fig. S5 (o, p). Note that in Fig. 1 of the main letter, we display quantum speed limits in frequency correlated systems rather than in frequency decorrelated systems.

S4.5 The relationship between the fidelity and the maximum quantum speed in multi-photon experiments

As the number of photons increases, the fidelity of the polarization state will decrease gradually. Here, we discuss the impact of the fidelity on the quantum speed using a simple model. In N-particle entangled systems, we assume that the initial polarization state is a depolarized state, mixing the optimal entangled initial states with the identity matrix:

$$\rho(0) = C_0 \left(|H\rangle^{\otimes N} + |V\rangle^{\otimes N}\right)\left(\langle H|^{\otimes N} + \langle V|^{\otimes N}\right)/2 + C_1 I/2^N. \tag{S63}$$

Here, $0 \leq C_0, C_1 \leq 1$, $C_0 + C_1 = 1$. When we use observable $A = \left(|H\rangle^{\otimes N} + |V\rangle^{\otimes N}\right)\left(\langle H|^{\otimes N} + \langle V|^{\otimes N}\right)/2$, the quantum speed at evolution time l is:

$$|\dot{a}(l)| = C_0 N\pi \left|\sin\left(2N\pi l/\lambda\right)\right|. \tag{S64}$$

The maximum quantum speed is $C_0 N\pi$. At the same time, the fidelity of the initial density matrix is:

$$\begin{aligned} F =& \left(\mathrm{Tr}\sqrt{\sqrt{\rho(0)}A\sqrt{\rho(0)}}\right)^2 \\ =& C_0 + C_1/2^N \\ =& \frac{1}{2^N} + \frac{2^N-1}{2^N}C_0. \end{aligned} \tag{S65}$$

So, the maximum quantum speed is:

$$\max_l |\dot{a}(l)| = \frac{2^N F - 1}{2^N - 1} N\pi. \tag{S66}$$

As shown in Fig. S6, the maximum quantum speed is almost proportional to the fidelity of the initial density matrix. The maximum quantum speed of many-body entangled states is consistently greater than that of few-body states within a wide range of fidelity. Due to the varying fidelity of multi-photon sources with different structures, it is necessary to comprehensively study how to implement experiments based on Fig. S4, S5, and S6.

S5 The wedge-shaped quartz crystals

We use four wedge-shaped quartz crystals to generate a variable-length quartz crystal system. As shown in Fig. S7, all wedge-shaped crystals have the same parameter of $M = 18$mm and $\alpha = 21.8°$. The distances between crystals are

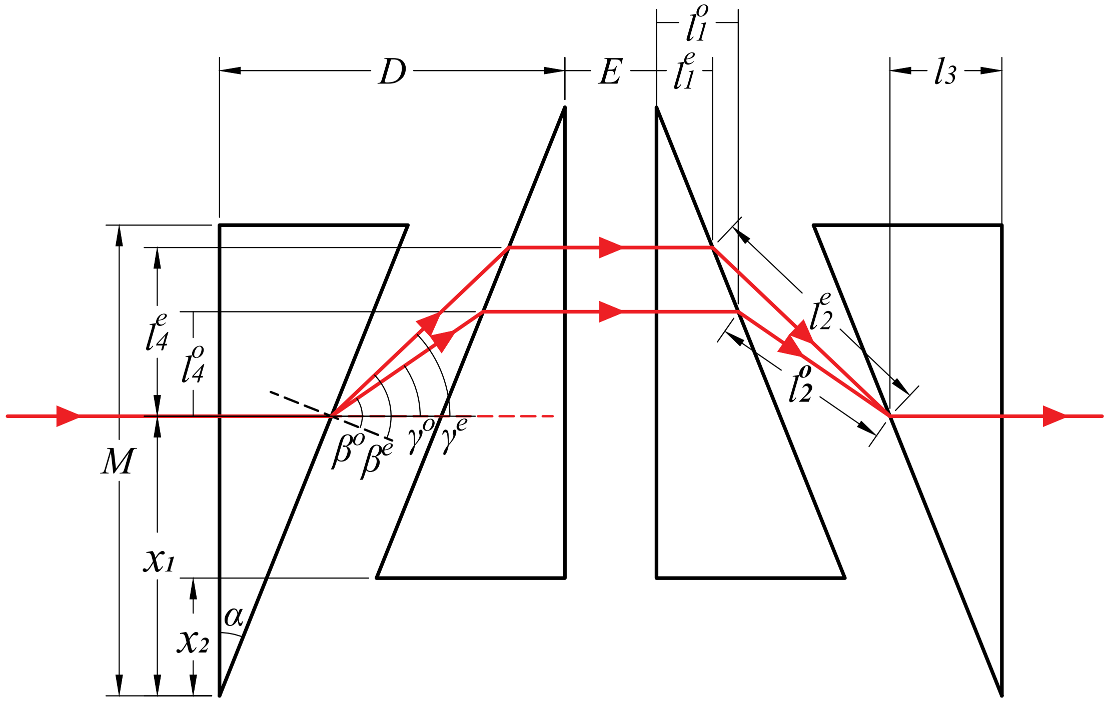

FIG. S7. **The optical path of ordinary light and extraordinary light in wedge-shaped quartz crystals.** x_2 represents the relative movement between two crystals in the middle and two crystals on the edge.

$D = 13.2$mm and $E = 5$mm. A light coming from the left side is perpendicularly incident on the quartz crystal, with the incident point located at x_1. For ordinary (o) light and extraordinary (e) light, they have different refractive indices (n_o and n_e), so they have different refractive angles, namely:

$$\beta^o = \arcsin\left(n_o \sin\alpha\right), \tag{S67}$$

$$\beta^e = \arcsin\left(n_e \sin\alpha\right), \tag{S68}$$

$$\gamma^o = \beta^o - \alpha, \tag{S69}$$

$$\gamma^e = \beta^e - \alpha. \tag{S70}$$

The middle two wedge-shaped quartz crystals are mounted on a linear motorized stage (LMS), allowing the horizontal movement as x_2. To allow the light to pass through four wedge-shaped quartz crystals, x_1 and x_2 must be within the range:

$$0 \leq x_1 \leq M, \tag{S71}$$

$$\frac{x_1 + D\frac{\cos\alpha\sin\gamma^e}{\cos(\alpha+\gamma^e)} - M\left[1 + \frac{\sin\alpha\sin\gamma^e}{\cos(\alpha+\gamma^e)}\right]}{\left[1 + \frac{\sin\alpha\sin\gamma^e}{\cos(\alpha+\gamma^e)}\right]} \leq x_2 \leq \frac{x_1 + D\frac{\cos\alpha\sin\gamma^o}{\cos(\alpha+\gamma^o)} - M\frac{\sin\alpha\sin\gamma^o}{\cos(\alpha+\gamma^o)}}{\left[1 + \frac{\sin\alpha\sin\gamma^o}{\cos(\alpha+\gamma^o)}\right]}. \tag{S72}$$

We name the distance of the light path in the second or the third crystal as l_1, that in the first or the fourth crystal as l_3, and that between the third and the fourth crystal as l_2. The o light and e light parallel propagate in the second to third crystals, with a horizontal offset of l_4 relative to the incident light.

$$l_1^o = -D\frac{\sin\alpha\sin\gamma^o}{\cos\left(\alpha+\gamma^o\right)} + M\frac{\sin\alpha\cos\gamma^o}{\cos\left(\alpha+\gamma^o\right)} - x_1\tan\alpha + x_2\frac{\sin\alpha\cos\gamma^o}{\cos\left(\alpha+\gamma^o\right)}, \tag{S73}$$

$$l_1^e = -D\frac{\sin\alpha\sin\gamma^e}{\cos\left(\alpha+\gamma^e\right)} + M\frac{\sin\alpha\cos\gamma^e}{\cos\left(\alpha+\gamma^e\right)} - x_1\tan\alpha + x_2\frac{\sin\alpha\cos\gamma^e}{\cos\left(\alpha+\gamma^e\right)}, \tag{S74}$$

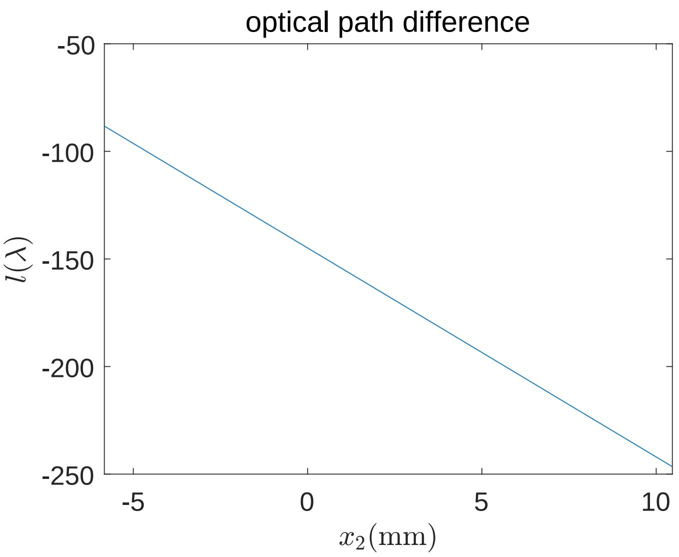

FIG. S8. **Optical path difference l vs horizontal movement x_2.**

$$l_2^o = D\frac{\cos\alpha}{\cos(\alpha+\gamma^o)} - M\frac{\sin\alpha}{\cos(\alpha+\gamma^o)} - x_2\frac{\sin\alpha}{\cos(\alpha+\gamma^o)}, \tag{S75}$$

$$l_2^e = D\frac{\cos\alpha}{\cos(\alpha+\gamma^e)} - M\frac{\sin\alpha}{\cos(\alpha+\gamma^e)} - x_2\frac{\sin\alpha}{\cos(\alpha+\gamma^e)}, \tag{S76}$$

$$l_3 = l_3^o = l_3^e = x_1\tan\alpha, \tag{S77}$$

$$l_4^o = D\frac{\cos\alpha\sin\gamma^o}{\cos(\alpha+\gamma^o)} - M\frac{\sin\alpha\sin\gamma^o}{\cos(\alpha+\gamma^o)} - x_2\frac{\sin\alpha\sin\gamma^o}{\cos(\alpha+\gamma^o)}, \tag{S78}$$

$$l_4^e = D\frac{\cos\alpha\sin\gamma^e}{\cos(\alpha+\gamma^e)} - M\frac{\sin\alpha\sin\gamma^e}{\cos(\alpha+\gamma^e)} - x_2\frac{\sin\alpha\sin\gamma^e}{\cos(\alpha+\gamma^e)}. \tag{S79}$$

The optical paths of o light and e light are:

$$l^o = n_o\left(2l_1^o + 2l_3\right) + 2l_2^o + E, \tag{S80}$$

$$l^e = n_e\left(2l_1^e + 2l_3\right) + 2l_2^e + E. \tag{S81}$$

As the optical axes of wedge-shaped quartz crystals are aligned in the $|V\rangle$ direction, the optical path difference between H polarization and V polarization is:

$$l = l^H - l^V \tag{S82}$$

$$\begin{aligned} = &+2D\left[+\frac{\cos\alpha}{\cos(\alpha+\gamma^o)} - \frac{\cos\alpha}{\cos(\alpha+\gamma^e)} - n_o\frac{\sin\alpha\sin\gamma^o}{\cos(\alpha+\gamma^o)} + n_e\frac{\sin\alpha\sin\gamma^e}{\cos(\alpha+\gamma^e)}\right] \\ &+2M\left[-\frac{\sin\alpha}{\cos(\alpha+\gamma^o)} + \frac{\sin\alpha}{\cos(\alpha+\gamma^e)} + n_o\frac{\sin\alpha\cos\gamma^o}{\cos(\alpha+\gamma^o)} - n_e\frac{\sin\alpha\cos\gamma^e}{\cos(\alpha+\gamma^e)}\right] \\ &+2x_2\left[-\frac{\sin\alpha}{\cos(\alpha+\gamma^o)} + \frac{\sin\alpha}{\cos(\alpha+\gamma^e)} + n_o\frac{\sin\alpha\cos\gamma^o}{\cos(\alpha+\gamma^o)} - n_e\frac{\sin\alpha\cos\gamma^e}{\cos(\alpha+\gamma^e)}\right]. \end{aligned} \tag{S83}$$

When we horizontally move the middle two wedge-shaped quartz crystals, we care about the change rate of l with the only independent variable x_2, namely:

$$\frac{\mathrm{d}l}{\mathrm{d}x_2} = 2\left[-\frac{\sin\alpha}{\cos(\alpha+\gamma^o)} + \frac{\sin\alpha}{\cos(\alpha+\gamma^e)} + n_o\frac{\sin\alpha\cos\gamma^o}{\cos(\alpha+\gamma^o)} - n_e\frac{\sin\alpha\cos\gamma^e}{\cos(\alpha+\gamma^e)}\right]. \tag{S84}$$

When the wavelength of the light is $\lambda = 808\text{nm}$, the change rate is $\frac{\mathrm{d}l}{\mathrm{d}x_2} = -9.7139\times10^{-3}\lambda/\mu\text{m} = -1\lambda/102.94\mu\text{m}$. As shown in Fig. S8, the 102.94μm movement results in the 1λ change in l. Perhaps there is a error of $+0.07°$ in the $\alpha = 21.8°$, in real experiment setup, the change rate is $\frac{\mathrm{d}l}{\mathrm{d}x_2} = -1\lambda/102.5\mu\text{m}$, still similar to the theoretical change rate.

We use SIGMA-KOKI TAMM100-50C LMS, which is driven by a 5-phase stepping motor with resolution 2μm/pulse(full). With the SHOT-702H controller, which can divide a full step to at most 250 division micro-step, our LMS can work using

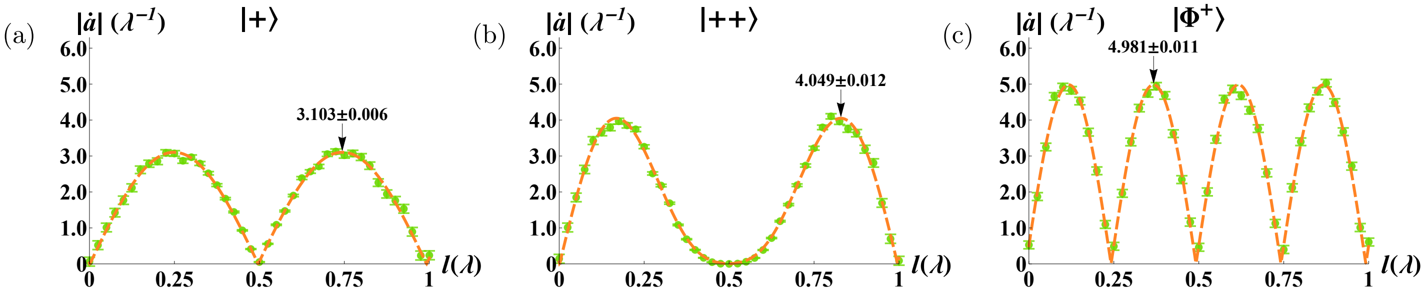

FIG. S9. **The fitted curves of the set of data points obtained from the experiments for the initial state of $|+\rangle$, $|++\rangle$, and $|\Phi^+\rangle$.** Green points represent quantum speeds in experiments. Orange lines represent the fitted curve.

20 division micro-step with resolution 0.1μm/pulse(micro). We can see a 0.1μm movement on x_2 results in approximately $9.7139\times10^{-4}\lambda \approx 0.001\lambda$ change in l. Here we achieve an accuracy up to 0.001λ in l.

The experimental setup is placed on a windproof, constant temperature, and humidity experimental platform; the environment is stable enough that there is no observable optical path difference shift within two days.

S6 The fitted curves

Our experimental results lie close to a curve. However, there are some fluctuations, which means that it's unreasonable to just use the greatest experimental data as the maximum quantum speed. Instead, we need to perform curve fitting and calculate the maximum value of the fitted curve.

In the single-particle qubit system, for the initial state of $|+\rangle$. Using Eq. (S28), the theoretical expectation value is $a=\frac{1}{2}\cos(2\pi l/\lambda)+\frac{1}{2}$ and the theoretical quantum speed is $|\dot{a}(l)|=|-\pi\sin(2\pi l/\lambda)|$. Therefore, we use the formula form $|-AB\sin(Bl/\lambda+C)|$ to fit the set of data points obtained from the experiment, shown in Fig. S9 (a). We perform 10,000 Monte Carlo samplings for every experiment; each sampling produces a fitted curve. The statistical fitting results are:

$$A = 0.4892 \pm 0.0007, \tag{S85}$$
$$B = 6.3432 \pm 0.0142, \tag{S86}$$
$$C = -0.0080 \pm 0.0077. \tag{S87}$$

Then the maximum quantum speed is:

$$|AB| = 3.103 \pm 0.006. \tag{S88}$$

In the product two-particle qubits system, for initial state of $|++\rangle$, the theoretical expectation value is $a=\left[\frac{1}{2}\cos(2\pi l/\lambda)+\frac{1}{2}\right]^2$ and the theoretical quantum speed is $|\dot{a}(l)| = |[\cos(2\pi l/\lambda)+1][-\pi\sin(2\pi l/\lambda)]|$. Therefore, we use the formula form $|-2[A\cos(Bl/\lambda+C)+D][AB\sin(Bl/\lambda+C)]|$ to fit experimental data, shown in Fig. S9 (b). The statistical fitting results are:

$$A = 0.4891 \pm 0.0016, \tag{S89}$$
$$B = 6.3183 \pm 0.0108, \tag{S90}$$
$$C = -0.0084 \pm 0.0062, \tag{S91}$$
$$D = 0.5118 \pm 0.0022. \tag{S92}$$

Then the maximum quantum speed is:

$$\frac{|B|\sqrt{|A|}\left(3|D|+\sqrt{8A^2+D^2}\right)\sqrt{4|A|+\frac{|D|\left(-|D|+\sqrt{8A^2+D^2}\right)}{|A|}}}{4\sqrt{2}} = 4.049 \pm 0.012. \tag{S93}$$

In the entangled two-particle qubits system, for initial state of $|\Phi^+\rangle$, the theoretical expectation value is $a=\frac{1}{2}\cos(4\pi l/\lambda)+\frac{1}{2}$ and the theoretical quantum speed is $|\dot{a}(l)|=|-2\pi\sin(4\pi l/\lambda)|$. Therefore, we use the formula form $|-AB\sin(Bl/\lambda+C)|$ to fit experimental data, shown in Fig. S9 (c). The statistical fitting results are:

$$A = 0.3963 \pm 0.0008, \tag{S94}$$
$$B = 12.5705 \pm 0.0099, \tag{S95}$$
$$C = 0.1018 \pm 0.0056. \tag{S96}$$

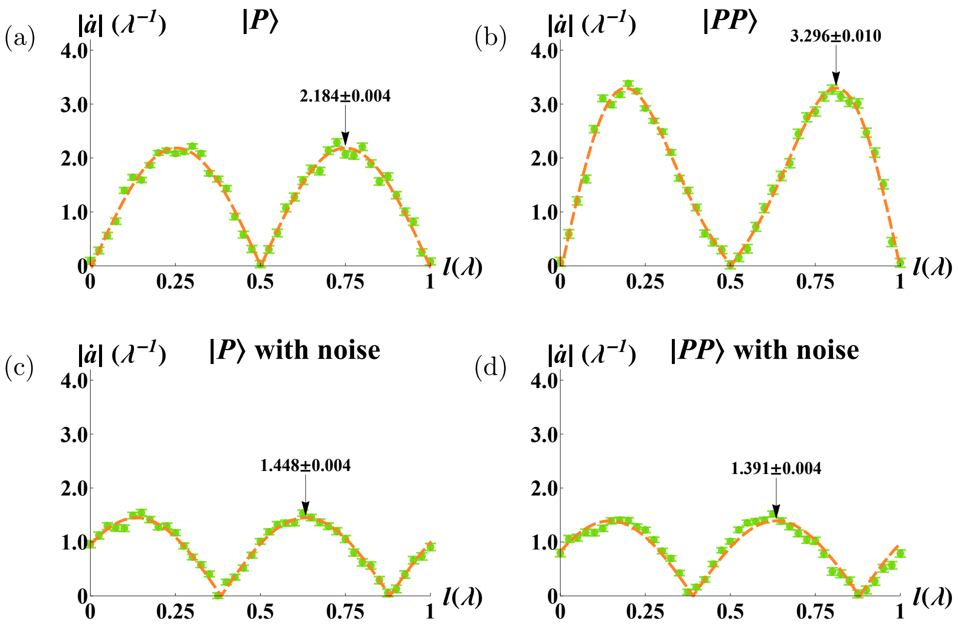

FIG. S10. **The fitted curves of the set of data points obtained from the experiments for the initial state of $|P\rangle$ without extra noise, $|PP\rangle$ without extra noise, $|P\rangle$ with extra noise and $|PP\rangle$ with extra noise.** Green points represent quantum speeds in experiments. Orange lines represent the fitted curve.

Then the maximum quantum speed is:

$$|AB| = 4.981 \pm 0.011. \tag{S97}$$

In the σ_x noisy conditions, as shown in Fig. S10, we explore quantum speed limits for the initial state of $|P\rangle$ and $|PP\rangle$ without and with extra noise.

For the initial state of $|P\rangle$ without extra noise. We use the formula form $|-AB\sin(Bl/\lambda + C)|$ to fit the set of data points obtained from experiments, shown in Fig. S10 (a). The statistical fitting results are:

$$A = 0.3452 \pm 0.0005, \tag{S98}$$
$$B = 6.3268 \pm 0.0105, \tag{S99}$$
$$C = -0.0316 \pm 0.0056. \tag{S100}$$

Then the maximum quantum speed is:

$$|AB| = 2.184 \pm 0.004. \tag{S101}$$

For the initial state of $|PP\rangle$ without extra noise. We use the formula form $|-2\left[A\cos(Bl/\lambda + C) + D\right]\left[AB\sin(Bl/\lambda + C)\right]|$ to fit the set of data points obtained from experiments, shown in Fig. S10 (b). The statistical fitting results are:

$$A = 0.3513 \pm 0.0023, \tag{S102}$$
$$B = 6.3228 \pm 0.0104, \tag{S103}$$
$$C = -0.0311 \pm 0.0057, \tag{S104}$$
$$D = 0.6685 \pm 0.0050. \tag{S105}$$

Then the maximum quantum speed is:

$$\frac{|B|\sqrt{|A|}\left(3|D| + \sqrt{8A^2 + D^2}\right)\sqrt{4|A| + \frac{|D|\left(-|D| + \sqrt{8A^2+D^2}\right)}{|A|}}}{4\sqrt{2}} = 3.296 \pm 0.010. \tag{S106}$$

For the initial state of $|P\rangle$ with extra noise. We use the formula form $|-AB\sin(Bl/\lambda + C)|$ to fit the set of data points obtained from experiments, shown in Fig. S10 (c). The statistical fitting results are:

$$A = 0.2289 \pm 0.0008, \tag{S107}$$
$$B = 6.3256 \pm 0.0161, \tag{S108}$$
$$C = 0.7091 \pm 0.0101. \tag{S109}$$

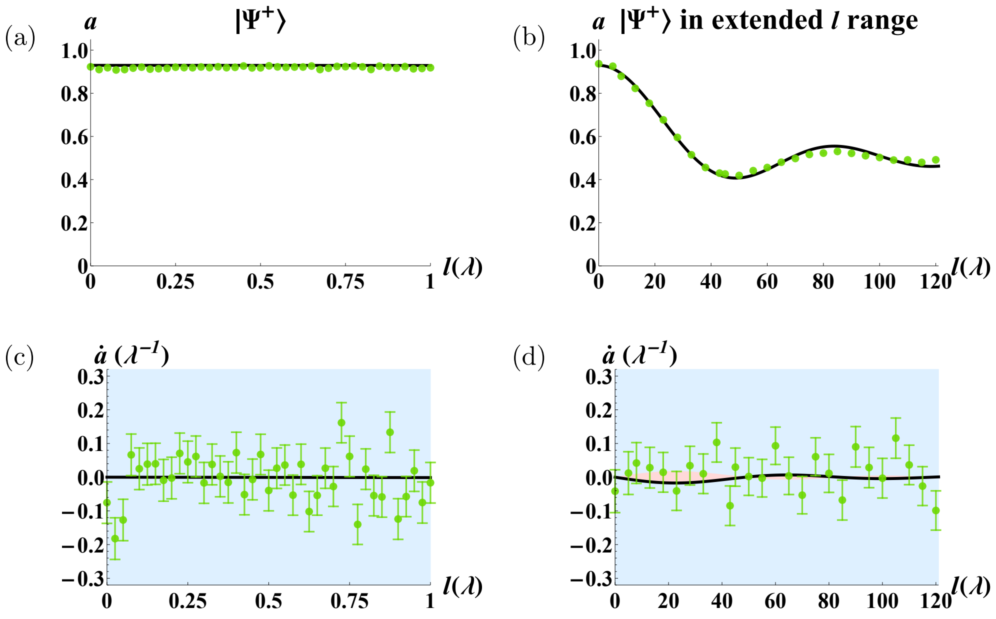

FIG. S11. **Quantum speed limits on observables of the initial state $|\Psi^+\rangle$. (a, b)** Expectation values a and on observables of the initial state. The black line and green points represent the expectation value a in theory and experiments. **(c, d)** Corresponding non-absolute quantum speeds $\dot{a}$ and speed limits on observables of the initial state. The black line and green points represent the non-absolute quantum speeds $\dot{a}$ in theory and experiments, respectively. The blue and red areas indicate the forbidden areas beyond the upper bound and below the lower bound, respectively. The initial state is $|\Psi^+\rangle$. The range of l is extended in (b, d). The upper bound, quantum speed, and lower bound are all almost zero during the entire evolution process.

Then the maximum quantum speed is:

$$|AB| = 1.448 \pm 0.004. \tag{S110}$$

For the initial state of $|PP\rangle$ with extra noise. We use the formula form $|-AB\sin(Bl/\lambda + C)|$ to fit the set of data points obtained from experiments, shown in Fig. S10 (d). The statistical fitting results are:

$$A = 0.2165 \pm 0.0008, \tag{S111}$$
$$B = 6.4256 \pm 0.0176, \tag{S112}$$
$$C = 0.6321 \pm 0.0110. \tag{S113}$$

Then the maximum quantum speed is:

$$|AB| = 1.391 \pm 0.004. \tag{S114}$$

S7 Entanglement can also decelerate quantum speed limits to almost zero

According to Eq. (S40), there are some near zero-speed states, such as $|\Psi^+\rangle$, that exhibit quantum speed, lower bounds, and upper bounds that are almost zero throughout the evolution, regardless of the observable. The only two additional phases $\exp\left[\mathrm{i}\frac{n_H(\omega_1)\omega_1 + n_V(\omega_2)\omega_2}{(\bar{n}_H - \bar{n}_V)c} l\right]$ and $\exp\left[\mathrm{i}\frac{n_V(\omega_1)\omega_1 + n_H(\omega_2)\omega_2}{(\bar{n}_H - \bar{n}_V)c} l\right]$ of $|HV\rangle$ part and $|VH\rangle$ part of initial state $|\Psi^+\rangle$, respectively. These two phases are almost equal to each other when the evolution time l changes because if one photon is $|H\rangle$ polarization, the other photon must be $|V\rangle$ polarization; which means certain forms of entanglement can balance the phase of two photons and decelerate quantum evolution speed to almost zero.

We experimentally investigate the tighter quantum speed limits with the initial state of $|\Psi^+\rangle$. The integral time for coincidence counting is 10 seconds, and the step of the slopes of the expectation values, namely Δl, is doubled to 0.050λ to decrease the jitter and the standard deviation. We still draw one point every 0.025λ. As shown in Fig. S11 (a), the expectation value remains nearly constant. The quantum speed is close to zero from $l = 0\lambda$ to $l = 1\lambda$. Therefore, we plot the non-absolute quantum speed $\dot{a}$ instead of quantum speed $|\dot{a}|$ in Fig. S11 (c) to make the figure clearer. For measuring the quantum speed in long-term evolution, we add two $+40\lambda$ quartz crystal plates before the wedge-shaped quartz crystals

in turn. Now the range of l is from 0λ to 120λ, and we draw a point every 5λ instead of 0.025λ to make the figure clearer. We observe no rapid periodic changes in the expectation value a but only slow dephasing between the different frequencies at almost zero speed, as shown in Fig. S11 (b, d). These results provide insights for the appropriate selection of specific robust quantum initial states to resist this type of noise.
